# Prognostic Biomarker TP53 Mutations for Immune Checkpoint Blockade Therapy and Its Association With Tumor Microenvironment of Lung Adenocarcinoma

**DOI:** 10.3389/fmolb.2020.602328

**Published:** 2020-11-19

**Authors:** Xinqing Lin, Liqiang Wang, Xiaohong Xie, Yinyin Qin, Zhanhong Xie, Ming Ouyang, Chengzhi Zhou

**Affiliations:** Department of Respiratory Medicine, The First Affiliated Hospital, Guangzhou Medical University, Guangzhou, China

**Keywords:** TP53, immune checkpoint inhibitors, tumor microenvironment, biomarker, mutation, prognosis

## Abstract

Immune checkpoint inhibitors (ICIs), is characterized by durable responses and improved survival in non-small cell lung cancer (NSCLC). However, there is a lack of predictive biomarkers to optimize the use of ICIs in cancers. The clinical benefit of patients with lung adenocarcinoma (LUAD) harboring TP53 mutations undergoing conventional treatments need to be optimized. Recently, studies indicated that TP53 mutations may be associated with improved survival in patients treated with ICIs. The immunotherapy cohort was used to estimate the association of TP53 mutations with the immune prognosis of LUAD. Genomic data were used to estimate the difference in immunogenicity and mutations in DNA damage repair (DDR). Clinical and genomic data were collected from patients with LUAD treated with ICIs and profiled using panel. The Cancer Genome Atlas (TCGA)-LUAD cohort was used to distinguish the tumor microenvironment, mutational profiles, immunogenicity and DDR mutations between TP53-mutated and TP53-wild-type. In the MSKCC-LUAD cohort, TP53-mutated LUAD showed significantly prolonged progression-free survival (PFS) (*P* = 0.017, *HR* = 0.69 [95%CI: 0.50–0.94]). CIBERSORT suggested that TP53-mutated had a higher proportion of activated immune cell infiltration. Additionally, TP53-mutated LUAD had higher expression levels of chemokines and proinflammatory mediators, increased tumor burden, neoantigen load, and DDR mutations. Gene set enrichment analysis (GSEA) suggests that TP53-mutated LUAD is significantly enriched in the cell cycle and DDR pathway but significantly downregulated in lipid metabolism. Our findings suggested that TP53 mutation may be a potential biomarker of immunotherapy for LUAD.

## Introduction

Non-small cell lung cancer (NSCLC) has been identified as the main type of lung cancer (approximately 85%), and lung adenocarcinoma (LUAD) is the most prevalent histologic type of NSCLC (approximately 60%) (Bray et al., 2018). The activation of TP53 mutations affecting the apoptosis pathway may result in NSCLC patients with TP53 mutations (while EGFR-wild-type) not responding well to platinum-based chemotherapy (Molina-Vila et al., 2014; Dong et al., 2017). Additionally, patients with EGFR-mutated NSCLC harboring TP53 mutations are often unlikely to derive clinical benefit (Molina-Vila et al., 2014; Canale et al., 2017; Zhang et al., 2019).

Immunotherapy, especially immune checkpoint inhibitors (ICIs), is characterized by durable responses and improved survival in a multitude of studies and trials, including advanced NSCLC (Brahmer et al., 2015; Horn et al., 2017). Specific biomarkers, such as PD-L1 expression, tumor mutation burden (TMB), blood TMB (bTMB), CD8+ T cell infiltration, immune signature and mismatch repair (MMR), are predictive biomarkers of patient benefit to ICIs (Dong et al., 2017; Wang et al., 2019). To date, studies have indicated that specific gene mutations can optimize the use of immunotherapy. Melanoma patients harboring SERPINB3/SERPINB4/NRAS mutations may have better clinical benefit from ICI treatment (Johnson et al., 2016; Riaz et al., 2016). Additionally, mutations involving important signaling pathways [DNA damage repair (DDR) (Rizvi et al., 2015) and IFN-γ (Zaretsky et al., 2016)] tend to predict immunotherapy benefits.

TP53 mutations are popular prognostic biomarkers, and researchers have been studying TP53 mutations in the prognosis of patients with LUAD. TP53 mutations can predict the clinical response of a combination of anti-CTLA-4 and anti-PD-(L)1 therapies in NSCLC (Hellmann et al., 2018b). Another group suggests that TP53 mutations, associated with high TMB, may be a potential biomarker for LUAD treated with anti-PD-(L)1 (Dong et al., 2017). We hypothesized that specific gene mutations in tumors may affect the tumor microenvironment (TME), such as upregulation of immune checkpoint expression, release of proinflammatory factors and chemokines, or recruitment of immune cells, ultimately affecting the efficacy and clinical prognosis of immunotherapy in patients (Topalian et al., 2016; Dong et al., 2017).

To confirm the above hypothesis and understand the molecular determinants of prognosis, we analyzed the differences between immune cells, proinflammatory cytokine chemokines, immunogenicity [such as TMB, neoantigen load (NAL) and DDR mutations] and signaling pathway activity between TP53-mutated and wild-type patients.

## Materials and Methods

### Identification of the Association Between TP53 Mutations and LUAD Immunotherapy Prognosis

To evaluate the association between TP53 mutations and the prognosis of LUAD patients treated with ICI treatment, we collected MSKCC-LUAD patients (*n* = 186) with clinical and mutation data (Rizvi et al., 2018). Patients with LUAD treated with anti-PD-(L)1 therapy were divided into TP53-mutated and TP53-wild-type for Kaplan-Meier (KM) analysis according to TP53 mutation (non-synonymous mutation). Additionally, TCGAbiolinks (Colaprico et al., 2016) R package was used to download the overall survival (OS) and mutation data. The cBioportal (Cerami et al., 2012) website tool was used to collect the disease-free survival (DFS) data.

### Characteristics of Genomic Mutations and Tumor Immunogenicity

Somatic mutation data (non-synonymous) of 186 MSKCC-LUAD patients were obtained from targeted next-generation sequencing (NGS). The NAL data of the TCGA-LUAD cohort were previously described (Thorsson et al., 2018). Consistent with other studies (Chalmers et al., 2017), the raw mutation count in TCGA-LUAD was divided by 38 Mb to quantify the TMB. The top 20 mutations and clinical characteristics of the MSKCC-LUAD and TCGA-LUAD cohorts were visualized using the R package ComplexHeatmap (Gu et al., 2016), and the mutation sites of TP53 in the MSKCC-LUAD and TCGA-LUAD cohorts were visualized using the R package Maftools (Mayakonda et al., 2018).

### Copy Number Variation (CNV) Analysis

The Affymetrix SNP 6.0 microarray data (hg19; germline/potential false-positive calls were removed) of the TCGA-LUAD cohort are available from the Broad GDAC Firehose^1^. GenePattern (Reich et al., 2006)^2^ was used to perform GISTIC 2.0 analysis on the downloaded copy number variation (CNV) segments and to identify the regions of significant gene amplification or deletion across the whole exome. The change in the number of focal somatic copies was determined with a 99% confidence level, and the X-chromosome was not excluded before analysis. The default settings were used except for the above parameters. The visualization module of the R package Maftools has easy-to-use and customizable features that help generate publication-quality images. We used the R package Maftools (Mayakonda et al., 2018) to visualize the analysis results above.

### Analysis of Immune Characteristics

CIBERSORT (Newman et al., 2015)^3^ with default parameters was used to analyze the expression data downloaded by TCGA biolinks to further estimate the contents of immune cells in LUAD. Additionally, we compared the mRNA expression levels of immune-related genes in TCGA-LUAD between TP53-mutated and TP53-wild-type samples, among which immune-related genes and immune-related signatures were identified (Thorsson et al., 2018). The expression levels of these genes were quantified as log2 (FPKM+1).

### Pathway Enrichment Analysis and DDR Analysis

The gene expression data of TCGA-LUAD were analyzed by using the R package edgeR (Robinson et al., 2010). Additionally, the clusterProfiler (Yu et al., 2012) R package was used for gene set enrichment analysis (GSEA), where the Gene Ontology (GO) terms, Kyoto Encyclopedia of Genes and Genomes (KEGG), and Reactome are thought to be significantly different in terms of *P* < 0.05. The DDR gene sets are from the MSigDB Database (Subramanian et al., 2005).

### Statistical Analysis

The Mann–Whitney *U-*test was used to compare the differences between TP53-wild-type and TP53-mutated TMB, NAL, immune cell abundance, immune-related gene expression, age, pack years, MSI score, and mutations in DDR pathways. Fisher’s exact test was used to compare the differences in the top 20 mutation statuses, genders, smoking histories, and responses between TP53-wild-type and TP53-mutated MSKCC-LUAD. The chi-square test was used to compare the differences in the top 20 mutation statuses, genders, ethnicities, smoking histories and clinical stages between TP53-wild-type and TP53-mutated patients in TCGA-LUAD. The log-rank test was used for the Kaplan-Meier (KM) analysis. A *P*-value < 0.05 was considered statistically significant, and all statistical tests were two-tailed. All statistical and visual analyses were carried out in R software (version 3.5.1). Additionally, the ComplexHeatmap R package was used to visualize the heatmap, and ggpubrwas (Kassambara, 2018) used to visualize the boxplot. The detailed analysis flow chart and sample size of this study are shown in Supplementary Figure S1.

## Results

### TP53 Mutations Are Associated With Better Immunotherapy Outcomes

**FIGURE 1:**
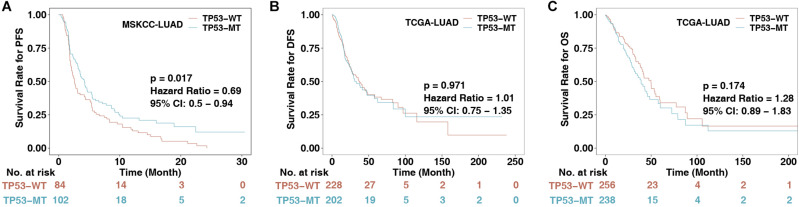
The association between TP53 status and clinical data. **(A)** KM survival analysis evaluated the relationship between TP53 status and PFS of patients in the MSKCC-LUAD cohort, *p* < 0.05. **(B)** Relationship between TP53 status and DFS of patients in the TCGA-LUAD cohort. DFS, disease-free survival. **(C)** Relationship between TP53 status and OS of patients in the TCGA-LUAD cohort. OS, overall survival. The log-rank test was used for a two-sided test, and *P* < 0.05 was regarded as significantly different.

To explore the association between TP53 mutations and immunotherapy prognosis in patients with LUAD, we collected patients from a published cohort (MSKCC-LUAD; *n* = 186). KM analysis was performed in LUAD patients divided according to TP53 status. The results showed that TP53-mutated LUAD patients had significantly longer PFS than TP53-wild-type LUAD patients (log-rank *P* = 0.017, HR = 0.69 [95% CI: 0.50–0.94]; Figure 1A). To explore the role of TP53 mutation in the non-immunotherapy cohort, the results suggested that there were no significant differences between TP53-mutated and TP53-wild-type LUAD patients in the TCGA-LUAD cohort in DFS (*P* = 0.971, HR = 1.01 [95% CI: 0.75–1.35]) and OS (*P* = 0.174, HR = 1.28 [95% CI: 0.89–1.83]; Figures 1B,C). This result indicates that the TP53 mutation may not predict the prognosis of the non-immunotherapy LUAD cohort.

### The Mutational Landscape of TP53-Mutated LUAD

**FIGURE 2:**
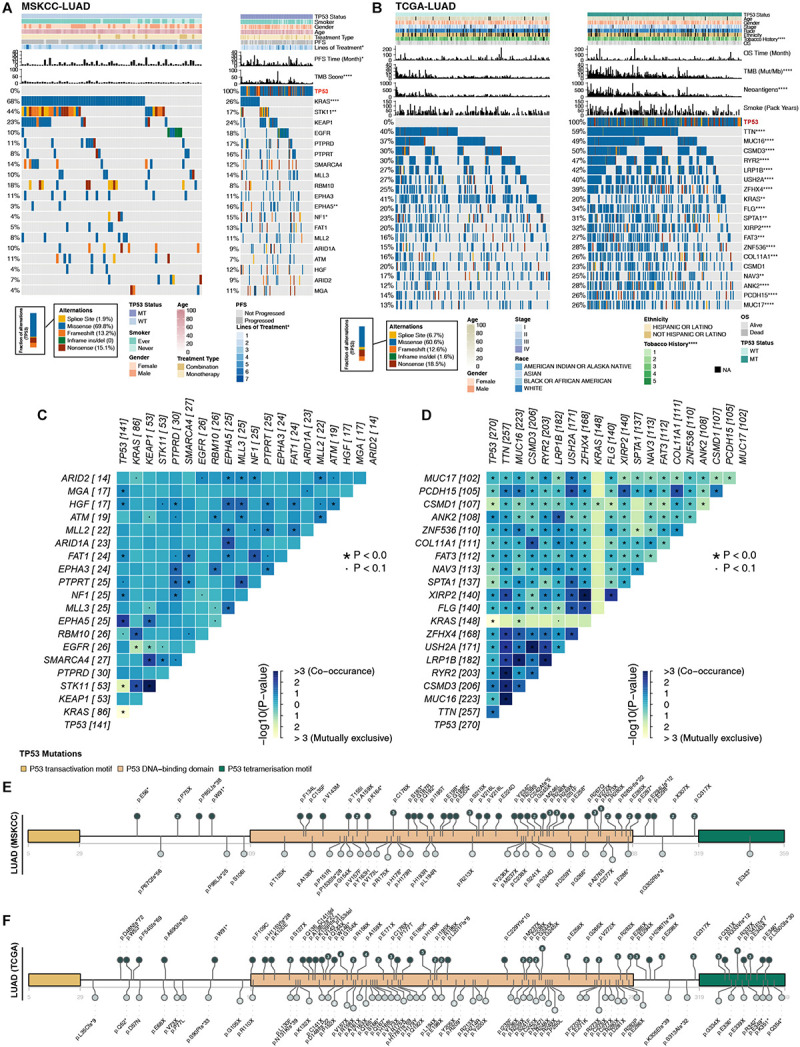
Comparison of related genetic characteristics and clinical data between TP53-mutated and TP53-wild-type LUAD. Panoramic views of TP53-wild-type and TP53-mutated patients in the MSKCC-LUAD cohort **(A)**, the TCGA-LUAD cohort **(B)**, including comparisons of genetic characteristics and clinical data. PFS, progression-free survival; OS, overall survival. Heatmap showing mutually exclusive and co-occurring mutations in the top 20 mutated genes in the MSKCC-LUAD cohort **(C)** and TCGA-LUAD cohort **(D)**. The mutation site of TCGA-TP53 in the MSKCC-LUAD cohort **(E)** and the TCGA-LUAD cohort **(F)**. Different colors correspond to different types of functions; the height of the circle represents the number of mutations. ****P < 0.0001, ***P < 0.001, **P < 0.01, *P < 0.05.

Higher immunogenicity is more easily recognized by the immune system of patients. First, in the three LUAD cohorts, TP53 had mostly missense and frameshift mutations (Figures 2A,B). Second, in the MSKCC-LUAD cohort (Figure 2A), the frequency of KRAS mutations in TP53-mutated cells was significantly lower than that in TP53-wild-type cells (26 vs. 68%; *P* < 0.0001; Fisher’s exact test). In the TCGA-LUAD cohort (Figure 2B), the TP53-mutated group had a lower KRAS mutation frequency (20 vs. 41%; *P* < 0.0001; Fisher’s exact test). Additionally, TP53-mutated and TP53-wild-type groups in MSKCC-LUAD with targeted sequencing have similar mutation frequencies in the top 20 mutations, except KRAS, STK11, EPHA5, and NF1. In contrast, compared with TP53-wild type, the mutation frequencies of the top 20 genes increased significantly in TCGA-LUAD, except for KRAS mutations (Figure 2B). Additionally, mutual exclusivity and co-occurrence analysis of the top 20 mutated genes in the MSKCC-LUAD and TCGA-LUAD cohort are shown in Figures 2C,D.

TP53 genes were mainly mutated in the P53 DNA–binding domain, while the P53 transactivation motif (N) and P53 tetramerization motif had fewer mutations (Figures 2E,F). In the two queues, most TP53 mutations were hotspot mutations (3D Hotspots database;^4^. Additionally, CNV analysis (GISTIC 2.0; Supplementary Figure S2) shows the differences in the levels of the chromosome arm in the TCGA-LUAD cohort and its subgroups (TP53-mutated and TP53-wild type). We found that in the TP53-mutated group, more fragments were amplified, such as 1q21.3 and 8q24.21. In addition, TP53-wild type on chromosome 1 had more missing piece, such as 1p21.3 and 1p13.2.

### TP53 Mutations Are Associated With Higher Immunogenicity and DDR Mutations

**FIGURE 3:**
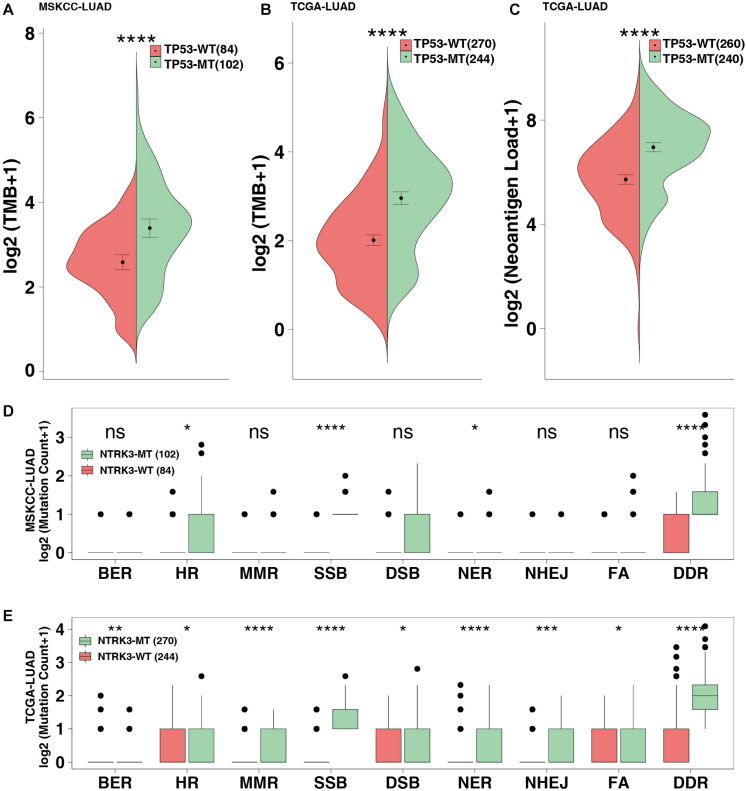
TP53 mutations improve the immunogenicity of tumors. TMB in the MSKCC-LUAD cohort **(A)** and the TCGA-LUAD cohort **(B)**. **(C)** Neoantigen loads (NAL) in the TCGA-LUAD cohort. The mutation count of DNA damage repair pathways in the MSKCC-LUAD cohort **(D)** and the TCGA-LUAD cohort **(E)**. BER, base excision repair; HR, homologous recombination; MMR, mismatch repair; NER, nucleotide excision repair; FA, Fanconi anemia pathways; SSB, single-stranded DNA binding; DSB, double-stranded DNA break repair; NHEJ, non-homologous end-joining. ****P < 0.0001, ***P < 0.001, **P < 0.01, *P < 0.05.

The MSKCC-LUAD cohort used targeted sequencing to quantify the TMB score, while the TCGA cohort used whole-exome sequencing (WES) to calculate the TMB and NAL. TP53-mutated LUAD significantly increased TMB in both the MSKCC-LUAD and TCGA-LUAD cohorts (*P* < 0.0001 and *P* < 0.00001, respectively, Figures 3A,B). Similarly, NAL in the TP53-mutated group was significantly higher than that in the TP53-wild-type group (*P* < 0.0001, Figure 3C).

The DDR pathway mainly includes mismatch repair (MMR), base excision repair (BER), nucleotide excision repair (NER), homologous recombination repair, non-homologous end-joining (NHEJ), Fanconi anemia (FA), double-strand break repair (DSB), and single-stranded DNA binding (SSB). The DDR gene sets (8 pathways from the MsiDB Database) were used in downstream analysis. DDR pathway analysis showed that the DDR mutations in the TP53-mutated MSKCC-LUAD significantly increased, including HR, SSB, NER and DDR (both *P* < 0.05; Figure 3D). Additionally, compared with TP53-wild type genes, TP53-mutated genes are likely to be genetically unstable (both *P* < 0.05; Figure 3E).

### TP53 Mutation Was Associated With Inflamed TME

**FIGURE 4:**
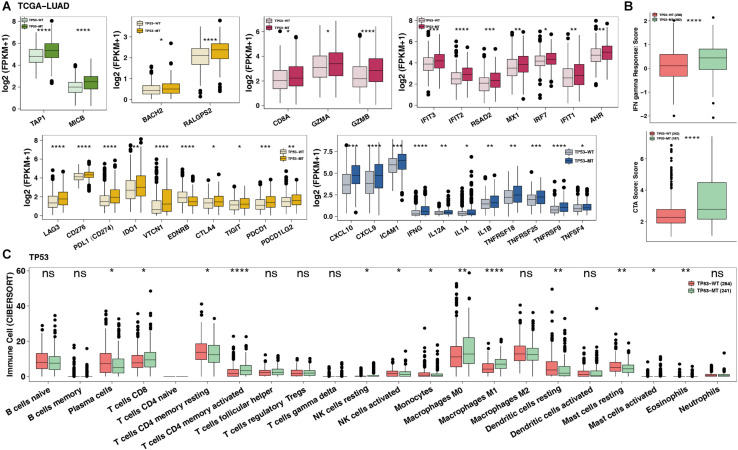
TP53 mutations mitigate immune resistance in LUAD. **(A)** The expression of immune-related genes (such as antigen presentation, B cells, cytolytic activity, immune inhibition, immune stimulation and IFN response) of TP53-mutated vs. TP53-wild type in TCGA-LUAD. **(B)** The immune score/signature of TP53-mutated vs. TP53-wild type in TCGA-LUAD. **(C)** The contents of immune cells of TP53-mutated vs. TP53-wild type in TCGA-LUAD using CIBERSORT analysis. ****P < 0.0001, ***P < 0.001, **P < 0.01, *P < 0.05.

To date, the crosstalk between immune cells and other cells or elements in the TME has been gradually recognized, and genomic changes may affect the components in the TME. Figure 4A shows that the TP53-mutated group had significantly increased expression levels of immune-related genes, such as potential antigen presentation (TAP1 and MICB), B cell-related (BACH2 and RALGPS2), cytolytic activity (CTA) (CD8A, GZMA, and GZMB), immune checkpoint-related (LAG3, CD276, CD274, IDO1, CTLA4, TIGHT, PDCD1, and PDCD1LG2), chemokine (CXCL9 and CXCL10), and proinflammatory mediators (interleukin, TNF, IFN-γ, and type I/II IFN response). Immune-related signature analysis indicated that TP53-mutated cells had higher IFN-γ and cytotoxic activity (CTA) scores (both *P* < 0.0001; Figure 4B). Additionally, CIBERSOTR showed that there were more activated macrophages in the TP53-mutated TME (Figure 4C), such as CD8+ T cells, activated CD8+ T cells, activated CD4+ memory T cells and M0/M1 macrophages (both *P* < 0.05). In contrast, resting CD4+ memory T cells were significantly enriched in the TP53-wild-type TME.

### Impact of TP53 Mutations on DNA Repair, Cell Cycle, DNA Replication, and Lipid Metabolism-Related Signaling

**FIGURE 5:**
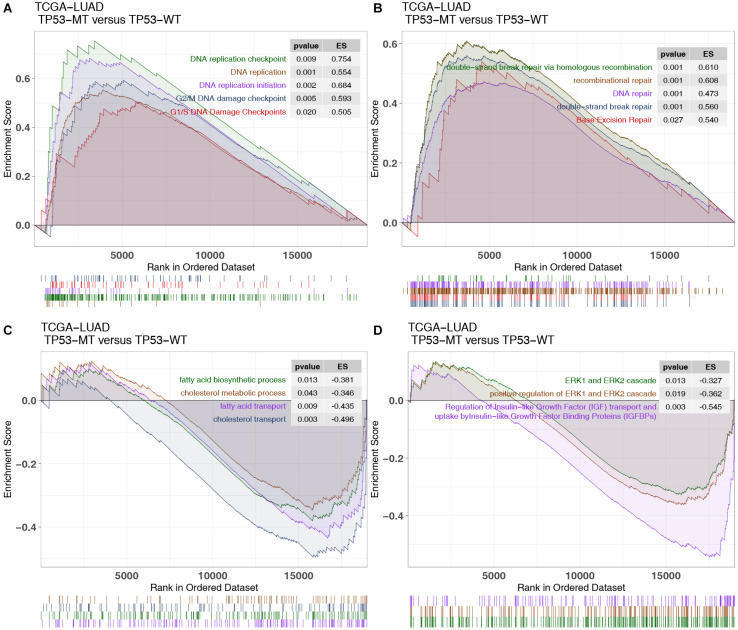
GSEA results and drug sensitivity analysis of TP53 mutations in the TCGA cohort. Part of the significant GSEA results of TP53-mutated patients: **(A)** The upregulated pathways in the GSEA results in the TCGA-LUAD cohort, including DNA replication-related pathways and G1/S (G2/M) DNA damage checkpoint pathways. **(B)** The upregulated pathways in the GSEA results in the TCGA-LUAD cohort, including DNA damage repair pathways. **(C)** The negatively regulated pathways in the GSEA results of the TCGA-LUAD cohort, including fatty acid and cholesterol metabolic pathways. **(D)** The negatively regulated pathways in the GSEA results of the TCGA-LUAD cohort, including oncogenic pathways.

There was an association between DNA repair, the cell cycle and DNA replication with immunogenicity in patients, while oncogenic and fatty metabolism may be associated with immune depletion. GSEA showed that TP53 mutations were significantly enriched in the DNA replication checkpoint [enrichment score (*ES*) > 0; *P* < 0.05; Figure 5A]. TP53-mutated LUAD shows enrichment in DDR pathways associated with genomic instability, including BER, DSB, and HR (Figure 5B). In contrast, some immune exhausted pathways (fatty acid biosynthesis/transport and cholesterol biosynthesis/transport) were significantly downregulated in the TP53-mutated group (*ES* < 0; *P* < 0.05; Figure 5C). Similarly, the activities of oncogenic signaling (such as ERK and IGFR) were significantly reduced in LUAD patients harboring TP53 mutations (Figure 5D).

## Discussion

**FIGURE 6:**
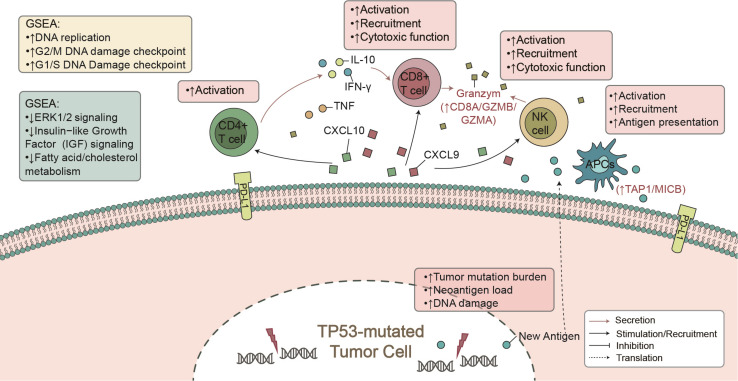
The mechanism of TP53 mutation affecting ICI prognosis in LUAD patients.

Here, from the perspective of TME, we found that TP53 mutations were associated with known biomarkers of immunotherapy (Lin et al., 2019), such as CD8+ T cell infiltration and IFN-γ and CTA signatures. Additionally, TP53-mutated LUAD showed higher immunogenicity, mainly manifested in the TMB, NAL, and DDR mutations. In the immune-cohort (MSKCC-LUAD), TP53-mutated LUAD was associated with better clinical prognosis (Figure 1A). These findings support our hypothesis that TP53 mutations represent a high degree of immunity and immunogenicity, as well as a state that inhibits the tumor’s own development (Figure 6).

To date, immune cells of TME have played a key role in clinical benefit from PD-1/PD-L1 blockade (Mariathasan et al., 2018). CD4+ and CD8+ T cells are recognized as antitumor immune cells by releasing INF-γ-, perforin-, and granzyme B-mediated cytotoxic effects. INF-γ plays an important role in raising and activating immune cells and then initiating the antitumor effect of proliferation and inducing tumor apoptosis (Fritzell et al., 2013; Garcia-Diaz et al., 2017). After contact with each other, T cells and tumor cells secrete CXCL9 and CXCL10, induced by INF-γ, with a positive feedback mechanism to increase the infiltration of immune cells (CD8+ T cells, NK cells, and macrophages) within the tumor (Van Allen et al., 2015; Tokunaga et al., 2018; Vitiello and Miller, 2020). Additionally, some studies reported that there was an association between better immunotherapy prognosis and increased immune-related gene expression profiles (GEPs), such as INF-γ and cytotoxic effect-related genes (CD8A, GZMA, GZMB, CD8B, etc.) (Jiang et al., 2018). In contrast, lipid metabolism plays a negative role in immunotherapy. For example, cholesterol binds to the T cell receptor (TCR) transmembrane region or disrupts the TCR signaling pathway, resulting in immune depletion of T cells (Swamy et al., 2016; Wang et al., 2016). Our GSEA results also suggest that TP53-mutated LUAD was significantly downregulated in fatty acid metabolism and transport, which often predicted tumor metastasis and development (Kim et al., 2019; Zhao et al., 2019). Consistent with other studies, high expression levels of immune checkpoints (such as: PDCD1, PDCD1LG2, CTLA4, LAG3) improved the prognosis of immunotherapy (Powles et al., 2014; Hugo et al., 2016; El-Khoueiry et al., 2017). Therefore, TP53 mutation may recruit and activate immune cells by secreting proinflammatory mediators and chemokines, upregulate immune-related gene expression, or downregulate lipid metabolism, ultimately increasing tumor killing activity.

High immunogenicity is conducive to the recognition of tumor cells by the immune system and further improves the clinical benefits of immunotherapy (Wang et al., 2019). TMB is a good biomarker for predicting the efficacy of ICIs and can quantitatively estimate the total number of mutations in the tumor genomic coding region (Van Allen et al., 2015; Hellmann et al., 2018a). Additionally, somatic mutations in the genome may produce tumor-specific antigens (such as neoantigens). It is generally believed that tumors with more mutations may produce more new epitopes that can be recognized by tumor-infiltrated T cells. The more mutations and NAL there are, the increased the risk of the immune system recognizing antigens, and immune treatment efficacy is better (Snyder et al., 2014; Wang et al., 2019). The somatic mutations in the DDR pathways mediated increased tumor genome instability. For example, DDR mutations can lead to loss of DNA repair activity and mediate the accumulation of incorrect DNA damage, indicating high TMB (Iyer et al., 2013; Teo et al., 2018). Moreover, antigen processing and presentation were critical factors affecting the efficiency of anti-PD-1/PD-L1 therapies (Mellman and Steinman, 2001). Effector T cells can activate STAT1 signaling to upregulate the expression of MHC-I on tumor cells by secreting INF-γ (Garcia-Diaz et al., 2017). Moreover, B cells, macrophages, and dendritic cells (DCs) transmitted the foreign peptide antigen to CD4+ T cells through MHC-II on the cell surface, thereby promoting tumor antigen recognition. The former can be specific to kill tumor cells, and the latter can secrete cytokines meditating tumor cell killing, playing an important role in antitumor positive feedback regulation (Lin et al., 2019). Here, the TP53-mutated group had a significant increase in TMB, NAL, and DDR mutations and immune-related GEP. Consistent with previous studies (Dong et al., 2017), TP53-mutated LUAD showed enrichment in the cell cycle, DDR, and DNA replication, leading to incorrect accumulation of DNA damage and genomic instability.

Our data indicate that T53-mutated LUAD is likely to benefit ICI therapy based on inflamed TME but that there may be certain limitations. First, due to panel sequencing of the MSKCC-LUAD cohorts, the number of mutations measured is significantly lower than that of the TCGA-LUAD cohort (WES). Second, the expression data of TCGA-LUAD are bulk-seq data, so the intratumor heterogeneity (like different functions of the group of immune cells) cannot be analyzed, but the bulk transcriptome data are only able to evaluate the average gene expression level and are unable to reveal the differences between immune cells. Third, this study used only the transcriptome and a portion of the genomics data to explain TP53 mutations as biomarkers of immunotherapy in LUAD patients. The future will still be based on omics prospective studies of large samples for subsequent analysis and verification.

## Conclusion

Based on the perspective of the TME, our study provides strong evidence that TP53 mutations are potential markers of ICI therapy in patients with LUAD. A large number of molecular mechanism and prospective clinical studies are still needed in the future to clarify the association between TP53 mutations and ICI treatment and to find hope for the survival of patients with LUAD.

## Data Availability Statement

All of the data we used in this study were publicly available as described in the Method section.

## Author Contributions

XL and CZ: conceptualization. XL, LW, and XX: formal analysis, software, visualization, writing–review and editing. YQ, ZX, MO, and CZ: supervision. XL, LW, XX, and MO: writing–original draft. All authors contributed to the article and approved the submitted version.

## Conflict of Interest

All authors have completed the ICMJE uniform disclosure form.

## Footnotes

1 http://gdac.broadinstitute.org/

2 https://www.genepattern.org/

3 http://cibersort.stanford.edu/

4 https://www.3dhotspots.org

## Supplementary Material

Supplementary Figure 1 **(A)** The workflow of the bioinformatics analysis. **(B)** Overlap of the efficacy-evaluable patient populations with assays used in this study.

Supplementary Figure 2 TP53 gene copy number variation map.
