# Supplementary material for: Prognostic Biomarker TP53 Mutations for Immune Checkpoint Blockade Therapy and Its Association With Tumor Microenvironment of Lung Adenocarcinoma
Source: Front Mol Biosci. 2020 Nov 19;7:602328. doi: 10.3389/fmolb.2020.602328 (PMC7710974; doi:10.3389/fmolb.2020.602328)
Supplement: Supplementary Figure 1 — (A) The workflow of the bioinformatics analysis. (B) Overlap of the efficacy-evaluable patient populations with assays used in this study. [file Data_Sheet_1.PDF]

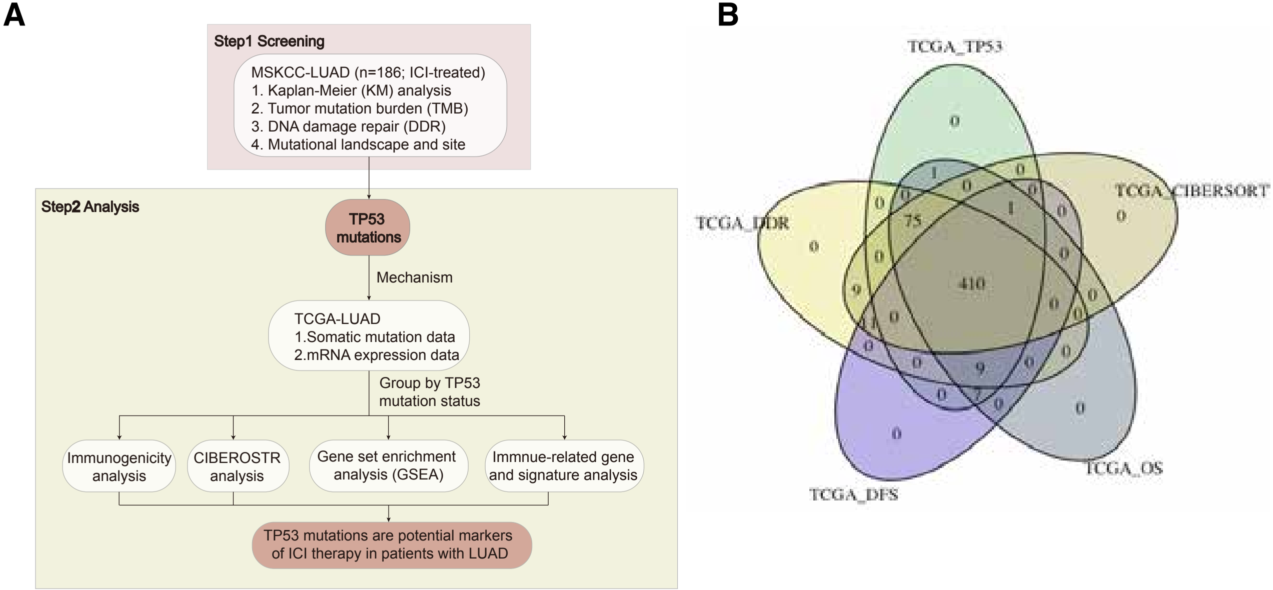
A
Step1 Screening
MSKCC-LUAD (n=186; ICI-treated)
1. Kaplan-Meier (KM) analysis
2. Tumor mutation burden (TMB)
3. DNA damage repair (DDR)
4. Mutational landscape and site
Step2 Analysis
TP53 mutations
Mechanism
TCGA-LUAD
1.Somatic mutation data
2.mRNA expression data
Group by TP53 mutation status
Immunogenicity analysis
CIBEROSTR analysis
Gene set enrichment analysis (GSEA)
Immnue-related gene and signature analysis
TP53 mutations are potential markers of ICI therapy in patients with LUAD
B
TCGA_TP53
TCGA_CIBERSORT
TCGA_DDR
TCGA_OS
TCGA_DFS
410
75
