# Supplementary figures and images for: Prognostic Biomarker TP53 Mutations for Immune Checkpoint Blockade Therapy and Its Association With Tumor Microenvironment of Lung Adenocarcinoma
Source: Front Mol Biosci. 2020 Nov 19;7:602328. doi: 10.3389/fmolb.2020.602328 (PMC7710974; doi:10.3389/fmolb.2020.602328)

# TCGA-LUAD

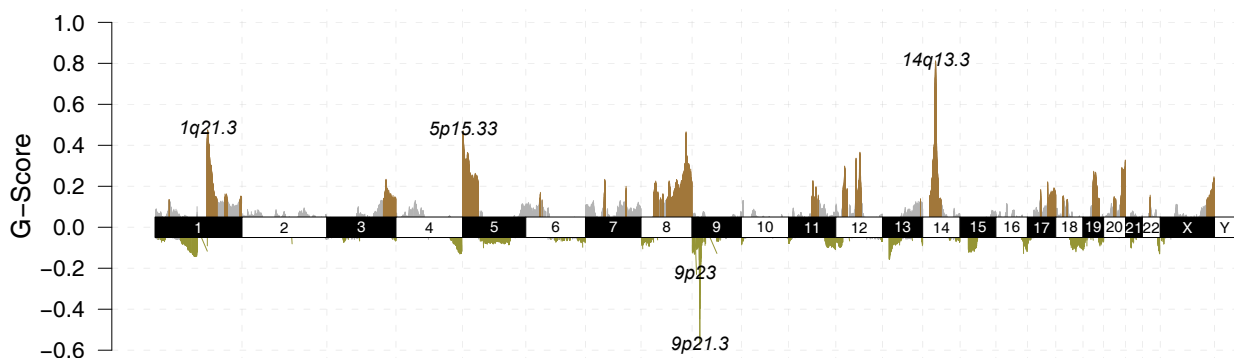

## TP53-MT (TCGA-LUAD)

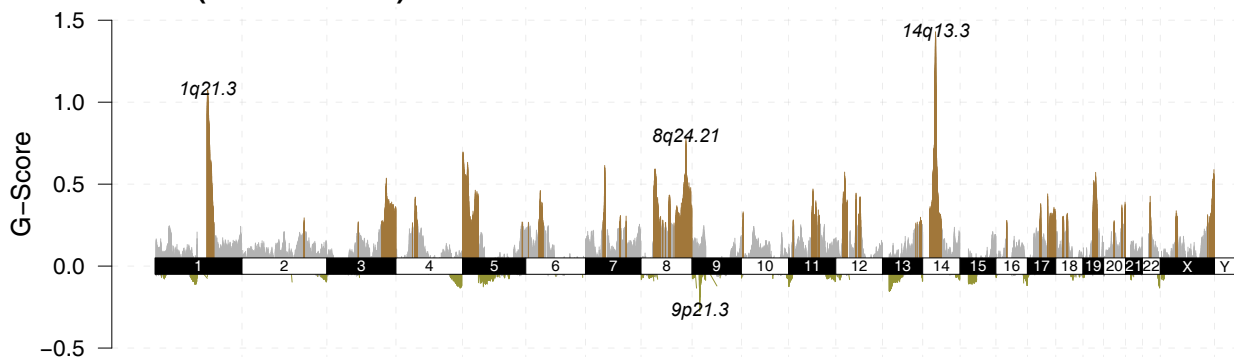

## TP53-WT (TCGA-LUAD)

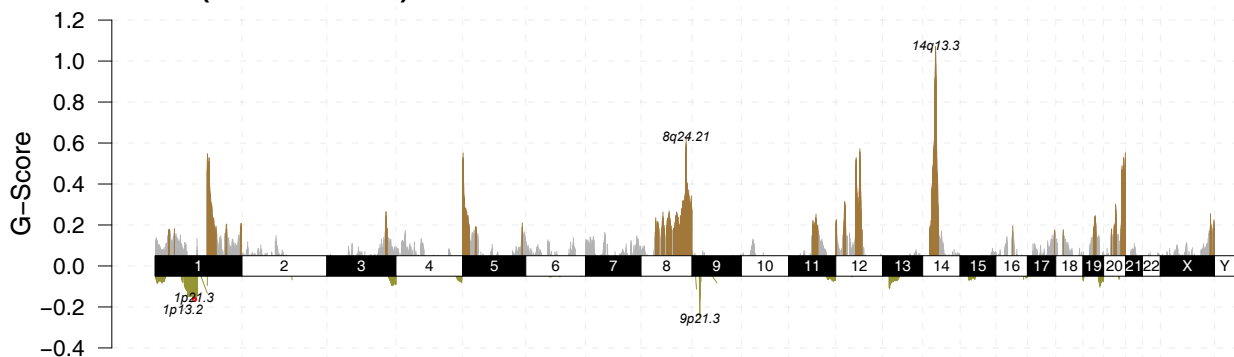

Supplement: Supplementary Figure 2 — TP53 gene copy number variation map. [file Data_Sheet_2.PDF]
